# Carascynol A, a hybrid of caryophyllane-type terpenoid and a C_6_ unit degraded by polyprenylated acylphloroglucinols from *Hypericum ascyron*

**DOI:** 10.1007/s13659-022-00362-z

**Published:** 2022-11-07

**Authors:** Ya-Li Hu, Xing-Ren Li, Gang Xu

**Affiliations:** 1 State Key Laboratory of Phytochemistry and Plant Resources in West China, Kunming Institute of Botany, Chinese Academy of Sciences, and Yunnan Key Laboratory of Natural Medicinal Chemistry, Kunming, 650201 Yunnan People’s Republic of China; 2 University of Chinese Academy of Sciences, Beijing, 100049 People’s Republic of China

**Keywords:** *Hypericum ascyron*, Caryophyllane, Polyprenylated acylphloroglucinols, Cytotoxicity, Colon cancer

## Abstract

**Abstract:**

Carascynol A, an unprecedented 4/9/8 ring system hybrid with a peroxide bridge, was characterized from *Hypericum ascyron*. The architecture contains a caryophyllane-type moiety and a C_6_ unit derived from polyprenylated acylphloroglucinols. Its structure and absolute configuration were determined by comprehensive spectroscopic and X-ray diffraction data. Biologically, compound **1** inhibited cell proliferation in LoVo, SW480, and HCT116 cell lines (IC_50_ = 12.30–24.57 µM).

**Graphical Abstract:**

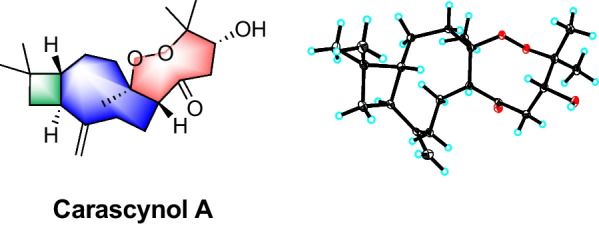

**Supplementary Information:**

The online version contains supplementary material available at 10.1007/s13659-022-00362-z.

## Introduction

Colorectal cancer (CRC) ranks as the second most lethal cancer and the third most prevalent malignant tumor worldwide [1]. Colon cancer, one of three types of CRC, accounts for the highest percentage of incidence and mortality rate [1, 2]. For cancer patients, surgery and chemotherapy are usually the first choices. Current chemotherapy includes single-agent therapy, mainly fluoropyrimidine (5-FU)-based, and multiple-agent regimens containing one or several drugs [3]. Due to chemical and biological diversity, natural products have always been a major source for pharmacotherapy, especially for cancer diseases [4].

**Fig. 1:**
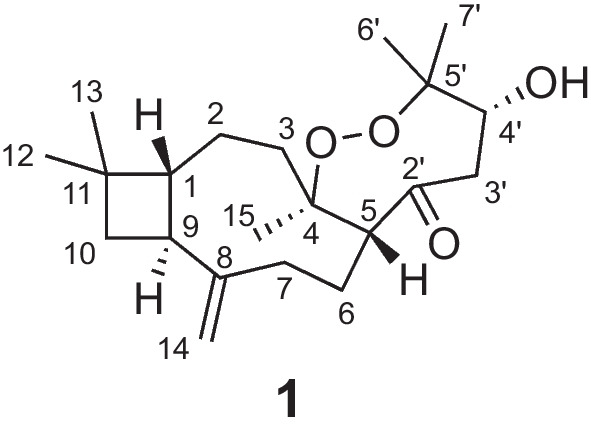
Structure of carascynol A (**1**)

Polycyclic polyprenylated acylphloroglucinols (PPAP), a special class of structurally diverse and biologically broad natural products, are rich in the plants of *Hypericum*. As one of the most widely distributed *Hypericum* species in China, *Hypericum ascyron* is a medicinal herb used in the treatment of abscesses and wounds [5]. Our previous studies on this plant have led to the characterization of some *seco*- and *nor*-PPAPs with anti-cancer activities [6, 7]. As a part of our systematic search for novel and bioactive natural PPAPs from *Hypericum* plants, further investigation on *H. ascyron* obtained an unprecedented hybrid condensed by a caryophyllane-type sesquiterpenoid and an uncommon C_6_ unit (Fig. 1). It is proposed that the C_6_ unit was derived from polyprenylated acylphloroglucinols by a cascade of ring-contracting isomerization, addition, and degradation reactions. Herein, the isolation, structure elucidation, plausible biosynthetic pathways, and biological evaluation of compound **1** were elaborated in this paper.

## Result and discussion

### Structure elucidation

Carascynol A (**1**) was obtained as colorless needles crystals. Its molecular formula C_21_H_34_O_4_ was established by its ^13^C NMR and HRESIMS data (m/z 373.2351, [M + Na]^+^, calcd for 373.2355), corresponding to 5 indices of hydrogen deficiency. The IR absorptions implied the presence of hydroxy (3430 cm^−1^), carbonyl (1712 cm^−1^), and terminal double-bond (3086, 1643, and 908 cm^−1^) groups. Its ^1^H NMR spectrum exhibited two olefinic protons (*δ*_H_ 4.82 and 4.96) and five methyl singlets (*δ*_H_ 0.96, 1.00, 1.01, 1.21, and 1.40). The ^13^C NMR and DEPT data presented a total of 21 carbon signals, including one non-conjugated carbonyl (*δ*_C_ 207.4), four quaternary carbons (including one unsaturated hydrocarbon at *δ*_C_ 151.7 and two oxygenated ones at *δ*_C_ 88.0 and 83.9), four methines (including an oxygenated one at *δ*_C_ 71.1), seven methylenes (including an olefinic one at *δ*_C_ 110.5), and five methylenes (Table 1).

**Table 1:** ^13^C (150 MHz) and ^1^H (600 MHz) NMR spectroscopic data of compound **1** in CDCl_3_

No.	*δ*_C_	*δ*_H_ (*J* in Hz)	No.	*δ*_C_	*δ*_H_ (*J* in Hz)
1	57.9, CH	1.66, m	11	34.6, C	
2a	22.6, CH_2_	1.64, m	12	29.9, CH_3_	1.00, s
2b		1.43, overlap	13	21.7, CH_3_	0.96, s
3a	39.6, CH_2_	1.81, m	14a	110.5, CH_2_	4.96, br s
3b		1.44, overlap	14b		4.82, br s
4	88.0, C		15	19.4, CH_3_	1.21, s
5	52.3, CH	2.79, dd (7.8, 3.0)	2′	207.4, C	
6a	25.6, CH_2_	1.99, m	3′a	51.0, CH_2_	2.55, dd (13.4, 5.5)
6b		1.59, m	3′b		2.51, dd (13.4, 11.2)
7a	36.2, CH_2_	2.10, m	4′	71.1, CH	4.64, m
7b		1.93, dd (10.8, 3.6)	5′	83.9, C	
8	151.7, C		6′	22.7, CH_3_	1.40, s
9	41.3, CH	2.42, q (9.7)	7′	16.6, CH_3_	1.01, s
10a	36.3, CH_2_	1.75, br t (10.5)			
10b		1.60, m			

**Fig. 2:**
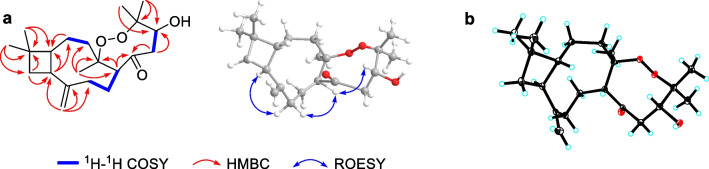
Key 2D NMR correlations (**a**) and ORTEP drawing (**b**) of **1**

The correlations from a gem-dimethyl at *δ*_H_ 1.00 (Me-12) and 0.96 (Me-13) to C-1 (*δ*_C_ 57.9)/C-10 (*δ*_C_ 36.3)/C-11 (*δ*_C_ 34.6), from H_2_-10 (*δ*_H_ 1.75 and 1.60) to C-9 (*δ*_C_ 41.3)/C-11, and from H-9 (*δ*_H_ 2.42) to C-1/C-10 in the HMBC spectrum, combined with the correlations of H-1 (*δ*_H_ 1.66)/H-9/H_2_-10 in the ^1^H-^1^H COSY spectrum, suggested the presence of a cyclobutane, which is characteristic for caryophyllene with 4/9-fused ring nucleus. Another nine-membered ring was established by the ^1^H-^1^H COSY correlations of H_2_-2 (*δ*_H_ 1.43 and 1.64)/H_2_-3 (*δ*_H_ 1.44 and 1.88) and H-5 (*δ*_H_ 2.79)/H_2_-6 (*δ*_H_ 1.59 and 1.99)/H_2_-7 (*δ*_H_ 1.93 and 2.10), together with the HMBC correlations from H_2_-2 to C-1, from H_2_-14 (*δ*_H_ 4.82 and 4.96) to C-1/C-7 (*δ*_C_ 36.2)/C-8 (*δ*_C_ 151.7)/C-9 (*δ*_C_ 41.3), and from Me-15 (*δ*_H_ 1.21) to C-3 (*δ*_C_ 39.6)/C-4 (*δ*_C_ 88.0)/C-5 (*δ*_C_ 52.3) (Fig. 2a).

Besides the existence of a caryophyllane-type sesquiterpenoid monomer, the remaining 6 carbons were connected by the correlations of H-5 to C-2′ (*δ*_C_ 207.4), H_2_-3′ (*δ*_H_ 2.51 and 2.55) to C-2′/C-4′ (*δ*_C_ 71.1), and Me-6′/7′ (*δ*_H_ 1.40 and 1.01) to C-4′/C-5′ (*δ*_C_ 83.9) in the HMBC spectrum. Additionally, a changeable proton and three additional oxygen atoms were unassigned in the molecular formula of **1**. Except for three unsaturated degrees attributed to the caryophyllane unit and one deficiency due to the carbonyl group, there should be one unsaturated degree left, which indicated that the C_6_ unit should be involved in further cyclization. We assumed that a peroxide bridge should lie between C-4 and C-5′ according to their apparently downfield chemical shifts at *δ*_C_ 88.0 and 83.9, respectively, while a hydroxyl group was located at C-4′ (*δ*_C_ 71.1). The single crystals of **1** [Flack parameter = − 0.09(10), CCDC 2212335] were obtained in methanol and subjected to an X-ray diffraction experiment with Cu K*α* radiation. The XRD result confirmed the planar structure (Fig. 2b).

In the ROESY spectrum (Fig. 2a), the cross-peaks of H-7b (*δ*_H_ 1.93)/H-5, H-5/H-4′ (*δ*_H_ 4.64), and H-7a (*δ*_H_ 2.10)/H-9 suggested that H-5 and H-4′ placed in the same orientation, while H-9 adopted the opposite orientation and was assigned as *α*. This deduction was consistent with the XRD result (Fig. 2b). Consequently, the absolute configuration of **1** was determined as 1*R*,4*R*,5*R*,9*S*,4′*R*.

**Scheme 1:**
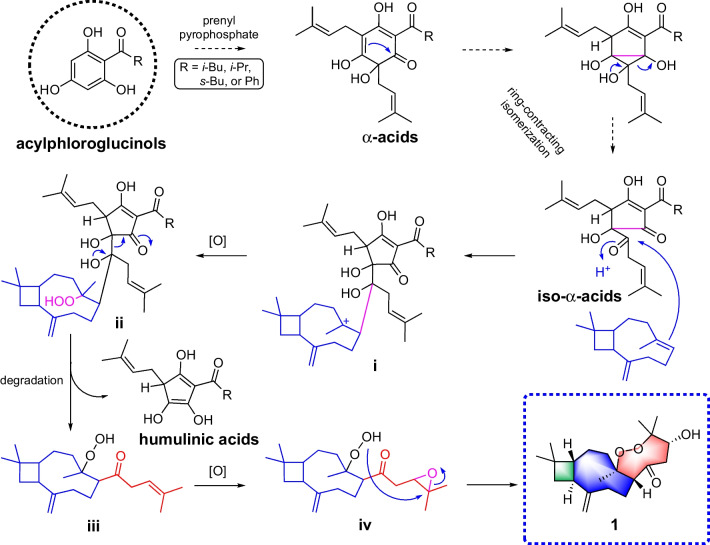
Plausible biosynthetic pathway to compound **1**

Polycyclic polyprenylated acylphloroglucinols (PPAP) possess highly oxygenated acylphloroglucinol-derived cores decorated with isoprenyl or geranyl side chains. Biosynthetically, prenylation of the acylphloroglucinols core moiety affords monocyclic polyprenylated acylphloroglucinols (MPAPs), which may be further cyclized to PPAP-type metabolites with diverse carbon skeletons [8–13]. In this study, we reckon that prenylation of acyphloroglucinols could also obtain polyprenylated acyphloroglucinols metabolites such as *α*-acids, which then naturally isomerized as its ring-contracted isomer (iso-*α*-acids) [14, 15]. Subsequently, iso-*α*-acids underwent an addition reaction with caryophyllene to form an intermediate **i**, which was oxidized to create a peroxide **ii**. Then, **ii** performed a degradation to produce **iii** and a humulinic acid [16]. Finally, the epoxidation of **iii** generated **iv**, which was cyclized to afford **1** (Scheme 1).

### Biological activity

The cytotoxicity of compound **1** against three human colon cancer cell lines (HCT116, SW480, and LoVo) was evaluated with fluorouracil (5-FU) as the positive control. As shown in Table 2, compound **1** showed the strongest activity against LoVo with IC_50_ values 12.30 ± 0.19 µM, while exhibited weaker cytotoxicity to SW480 and HCT116 cell lines (IC_50_ 18.33 ± 1.68 and 24.57 ± 3.09 µM). Compound **1** did not significantly alter the viability of PBMCs, suggesting its selective cytotoxicities on colon cancer cells.

**Table 2:** Cytotoxicity of compound **1**

	IC_50_ (µM)^a^		
	Cell lines	**1**	5-FU^b^
MTT assay	LoVo	12.30 ± 0.19	26.63 ± 1.05
	SW480	18.33 ± 1.68	81.30 ± 6.64
	HCT116	24.57 ± 3.09	22.12 ± 2.57
	PBMC	> 40	–

^a^Data were expressed as mean ± SD (n ≥ 3)

^b^5-FU was used as a positive control

## Conclusion

Actually, the hybridization of acylphloroglucinols core and *β*-caryophyllene unit has been described previously [9, 17]. However, it is the first time that the condensation of a sesquiterpenoids unit with an unusual C_6_ polyprenylated acylphloroglucinols degraded moiety in *Hypericum* species has been reported. Biogenetically, *β*-caryophyllene and polyprenylated acylphloroglucinols conjugated through a nucleophilic addition to produce a key intermediate, which upon multistep degradation, oxidation, and cyclization afforded **1**. Besides, our study revealed that compound **1** exhibited cytotoxicities on LoVo, SW480, and HCT116, with IC_50_ values in the range of 12.30–24.57 µM. To sum up, the present study may provide a new perspective for the structural and biological explorations of the terpenoid and PPAP hybrids.

## Supplementary Information

**Additional file 1.** The details of isolation and biological experimental procedures, physical and crystal data, and original NMR and MS spectra.
