# Supplementary material for: Carascynol A, a hybrid of caryophyllane-type terpenoid and a C6 unit degraded by polyprenylated acylphloroglucinols from Hypericum ascyron
Source: Nat Prod Bioprospect. 2022 Nov 7;12(1):38. doi: 10.1007/s13659-022-00362-z (PMC9640506; doi:10.1007/s13659-022-00362-z)
Supplement: Supplementary file 1 — Additional file 1. The details of isolation and biological experimental procedures, physical and crystal data, and original NMR and MS spectra. [file 13659_2022_362_MOESM1_ESM.pdf]

Carascynol A, a hybrid of caryophyllane-type terpenoid and a C_6 unit degraded by polyprenylated acylphloroglucinols from *Hypericum ascyron*

Ya-Li Hu, Xing-Ren Li, and Gang Xu

Supplementary Information

Table of contents

- SI-1. Experimental section (Page S2–S3)
- SI-2. Physical data of compound **1** (Page S4)
- SI-3. Crystal data for compound **1** (Page S4)
- SI-4. The original NMR and MS spectra of compound **1** (Page S5–S8)

SI-1. Experimental section

1.1 General experimental procedures

Optical rotation was measured on a Jasco P-1020 polarimeter with MeOH as solvent. UV spectrum was recorded on a Shimadzu UV-2401PC spectrometer. IR spectrum was determined by using a Bruker FT-IR Tensor-27 infrared spectrophotometer with KBr disk. 1D and 2D NMR spectra were recorded on a Bruker AV III-600 spectrometer. ESIMS and HRESIMS data were acquired on Agilent G6230 TOF mass spectrometer. Semi-preparative HPLC was performed on an Agilent 1100 HPLC with a Zorbax SB-C_{18} (9.4 × 250 mm) column. Silica gel (100-200 and 200-300 mesh, Qingdao Marine Chemical Co., Ltd., Qingdao, People's Republic of China), and MCI gel (75–150 μm, Mitsubishi Chemical Corporation, Tokyo, Japan) were used for column chromatography. Fractions were monitored by TLC (GF 254, Qingdao Marine Chemical Co., Ltd.), and spots were visualized by heating silica gel plates immersed in 10%H_2SO_4 in ethanol.

1.2 Plant material

The aerial parts of *Hypericum ascyron* were collected in Haba Snow Mountain of Shangri-La, Yunnan Province, P. R. China, in August 2015. The plant was identified by Dr. Yong-Zeng Zhang, Kunming Institute of Botany, Kunming, P. R. China. A voucher specimen was deposited with Kunming Institute of Botany with identification number 2015H01.

1.3 Extraction and isolation

The air-dried and powdered entire plants of *H. ascyron* (16.0 kg) were extracted with MeOH at room temperature and then filtered. The solvent was evaporated in vacuo and the obtained crude extract (5.0 kg) was subjected to a silica gel column chromatography eluted with petroleum ether/acetone in gradient (1:0-1:1, v/v) to afford five fractions (Fr. A–E). Fraction C (63 g) was separated over an MCI-gel column (MeOH-H_2O from 6:4 to 10:0, v/v) to obtain five fractions (Fr. C1–C5). Fraction C4 (5.2 g) was further chromatographed over a silica gel column, eluted with petroleum ether/acetone (200:1-0:1, v/v), to obtain six fractions (Fr. C4.1-C4.6). Compound **1** (3.6 mg) was purified from Fr. C4.4 (330 mg) by preparative HPLC.

1.4 Biological assay

Human colon cancer cell lines (HCT116, SW480, LoVo) were purchased from American Tissue Culture Collection (VA, USA). HCT116 cells were cultured in McCoy' s 5A medium supplemented with 10%v/v fetal bovine serum (FBS) and 1% penicillin/streptomycin (PS), while SW480 and LoVo cells were maintained in DMEM medium with 10%v/v FBS and 1% PS. All the cell lines were incubated at 37°C in a humidified atmosphere containing 5% CO_2. All culture medium and supplements were purchased from Thermo Fisher Scientific (MA, USA). Human peripheral blood mononuclear cells (PBMCs) were isolated from human buffy coat preparations obtained from the Hong Kong Red Cross Blood Transfusion Service. Chemicals such as 3-(4,5)-dimethylthiahiazo(-z-y1)-3,5-di-phenytetrazoliumromide (MTT) were obtained from Sigma-Aldrich (Merck KGaA, Germany).

Cell viability was assessed by MTT assay. Cells (5×10^4 cells/ mL) were seeded in 96-well flat-bottom culture plate in 100 μL of medium and incubated at 37°C overnight before testing compounds were added onto the cells for an incubation for 48 h. PBMCs (3×10^6 cells/mL) were seeded in 96-well culture plate and incubated with testing compounds for 48 h.

SI-2. Physical data of compound 1

*Carascynol A (1)***:** colorless needle crystals; mp 152–155 °C; $[\alpha]^{18}_{D}$ –64 (*c* 0.1, MeOH); UV (MeOH) λ_{max} (log ε) 195 (2.97), 280 (1.24) nm; IR (KBr) ν_{max} 3430, 3086, 1712, 1643, 1447, 908, 689 cm^{-1}; ECD (MeOH) λ_{max} ($\Delta\varepsilon$) 290 (+0.6), 223 (–0.4), 200 (+1.7); ^{1}H and ^{13}C NMR data, see Table 1; HRESIMS *m/z* 373.2351 $[M + Na]^+$ (calcd for $C_{21}H_{34}O_4Na$, 373.2355).

SI-3. Crystal data for compound 1

$C_{21}H_{34}O_4$, M = 350.48, a = 6.2381(2) Å, b = 14.3663(4) Å, c = 22.2682(5) Å, α = 90°, β = 90°, γ = 90°, V = 1995.64(10) $Å^3$, T = 100(2) K, space group P212121, Z = 4, μ(CuKα) = 0.627 mm^{-1}, 12374 reflections measured, 3639 independent reflections (R_{int} = 0.0509). The final R_1 values were 0.0411 ($I > 2\sigma(I)$). The final $wR(F^2)$ values were 0.0970 ($I > 2\sigma(I)$). The final R_1 values were 0.0438 (all data). The final $wR(F^2)$ values were 0.0984 (all data). The goodness of fit on F^2 was 1.049. Flack parameter = -0.09(10).

SI-4. The original NMR and MS spectra of compound 1

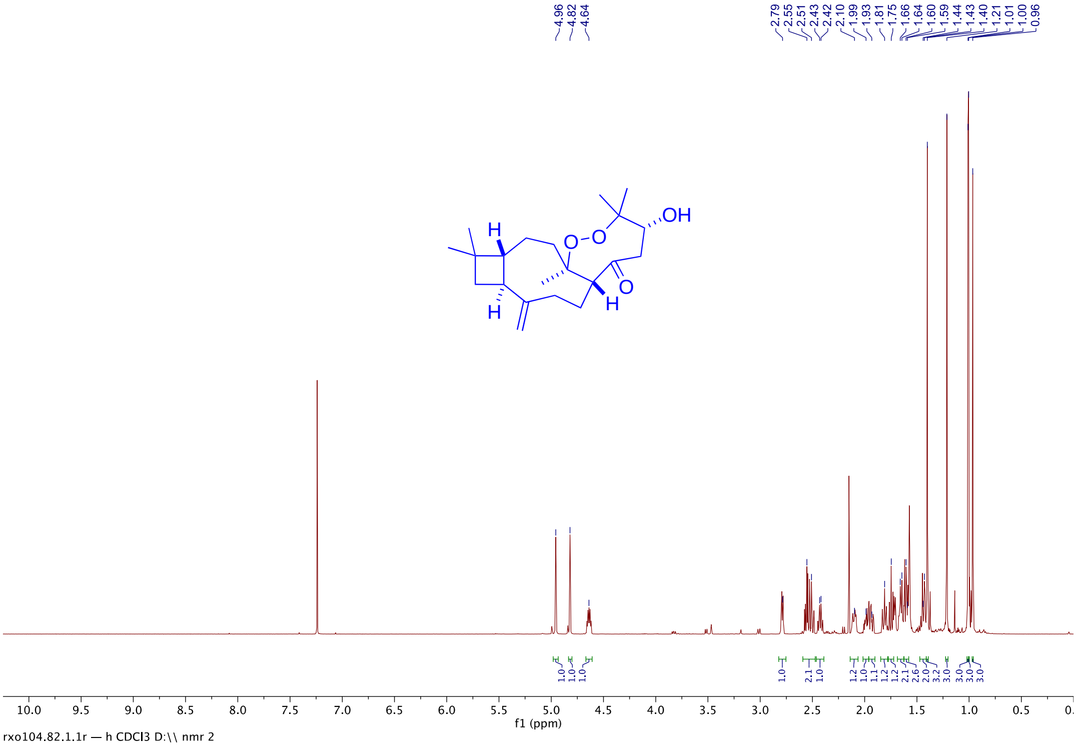

Figure S1. ^{1}H (in CDCl$_3$) spectrum of carascynol A (**1**).

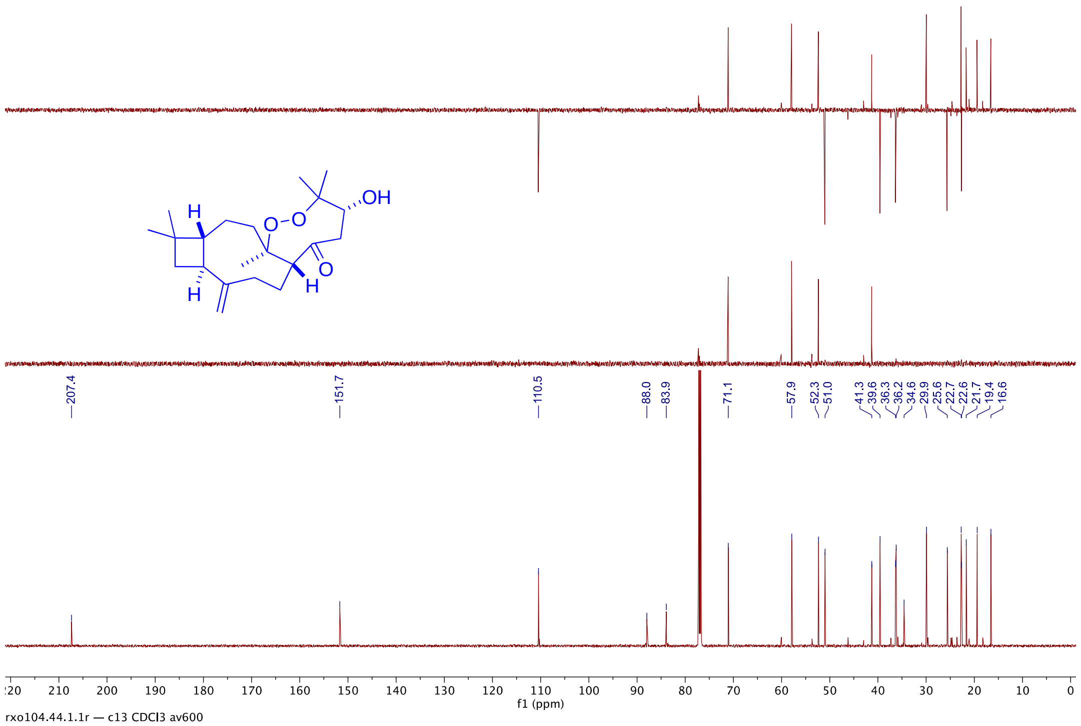

Figure S2. ^{13}C and DEPT (in CDCl$_3$) spectra of carascynol A (**1**).

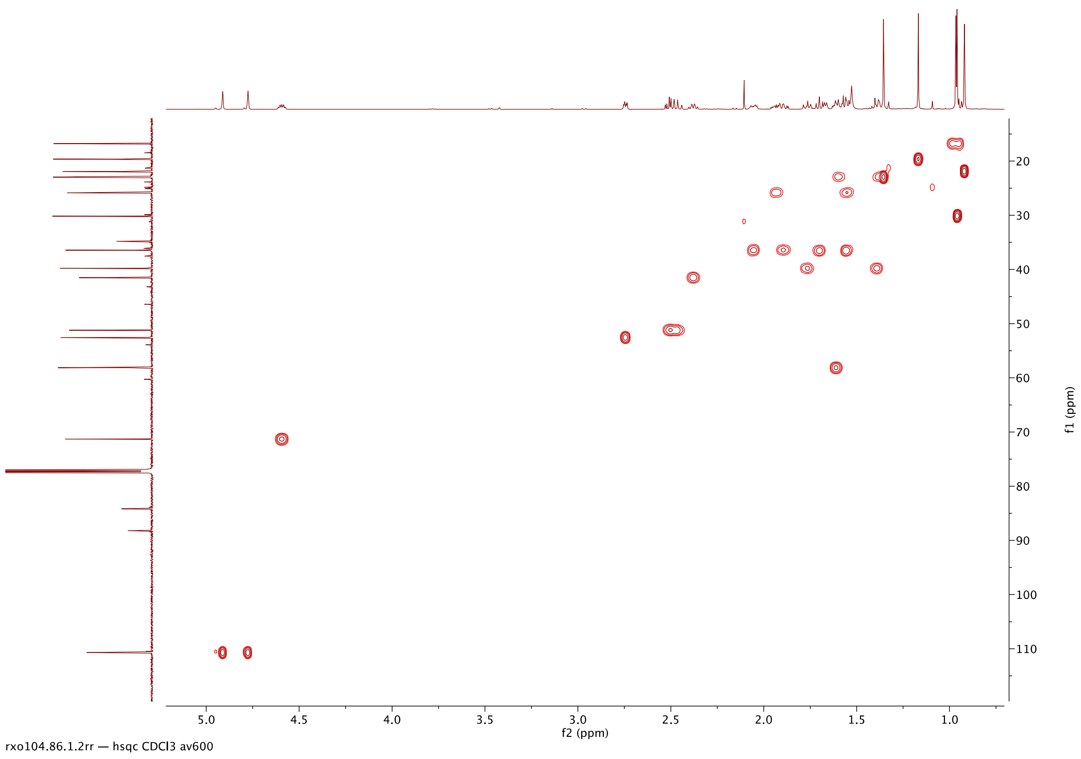

Figure S3. HSQC spectrum of carascynol A (**1**).

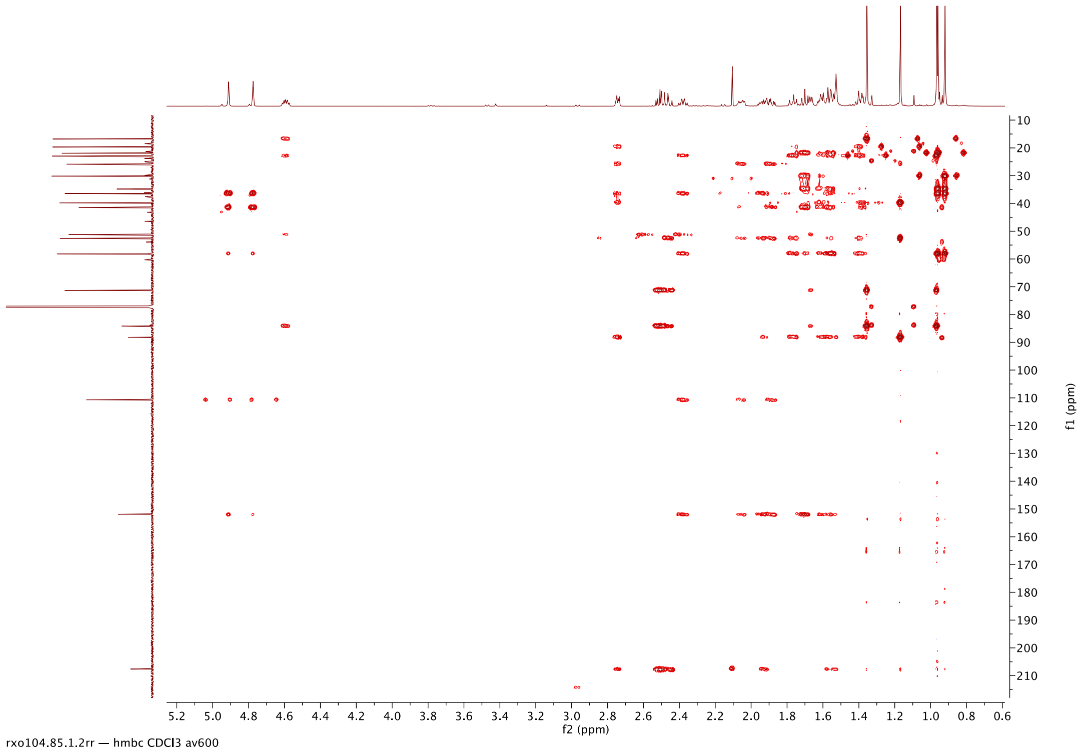

Figure S4. HMBC spectrum of carascynol A (**1**).

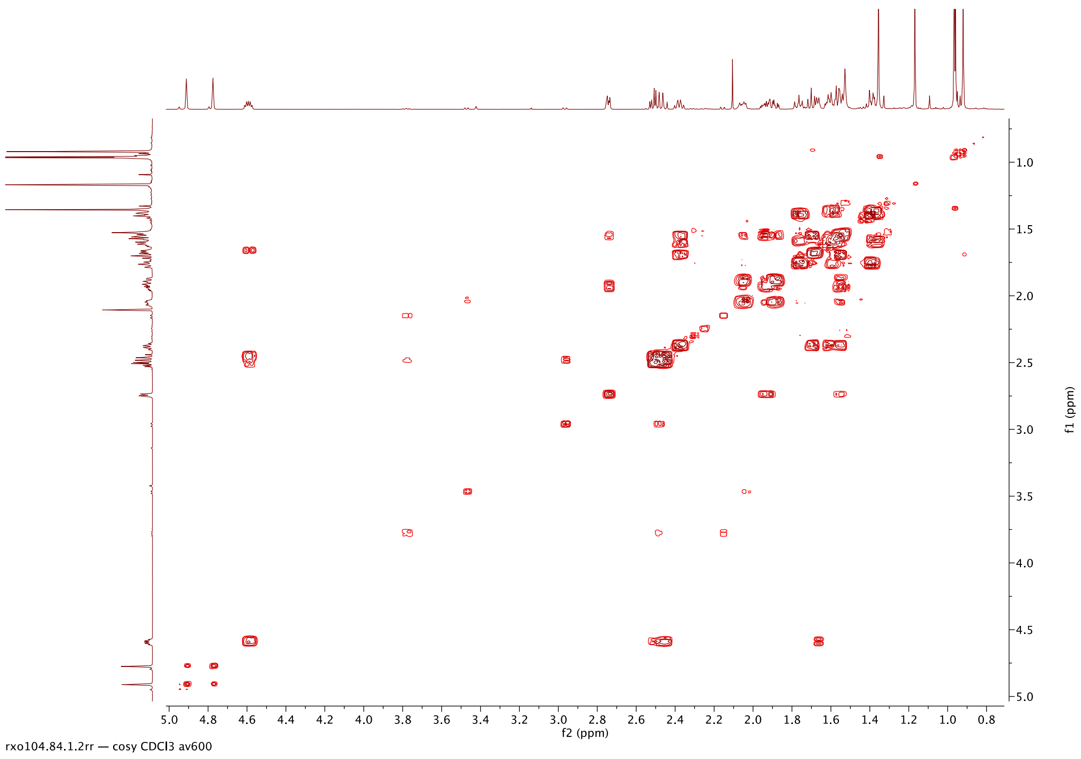

Figure S5. ^{1}H–^{1}H COSY spectrum of carascynol A (**1**).

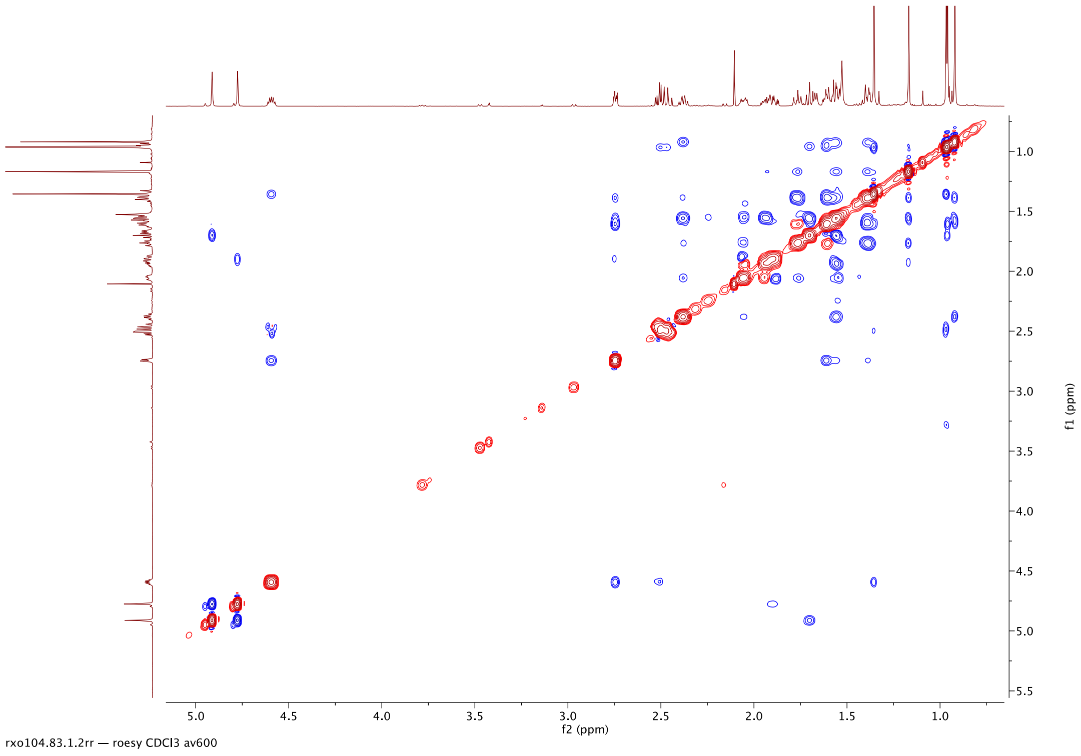

Figure S6. ROESY spectrum of carascynol A (**1**).

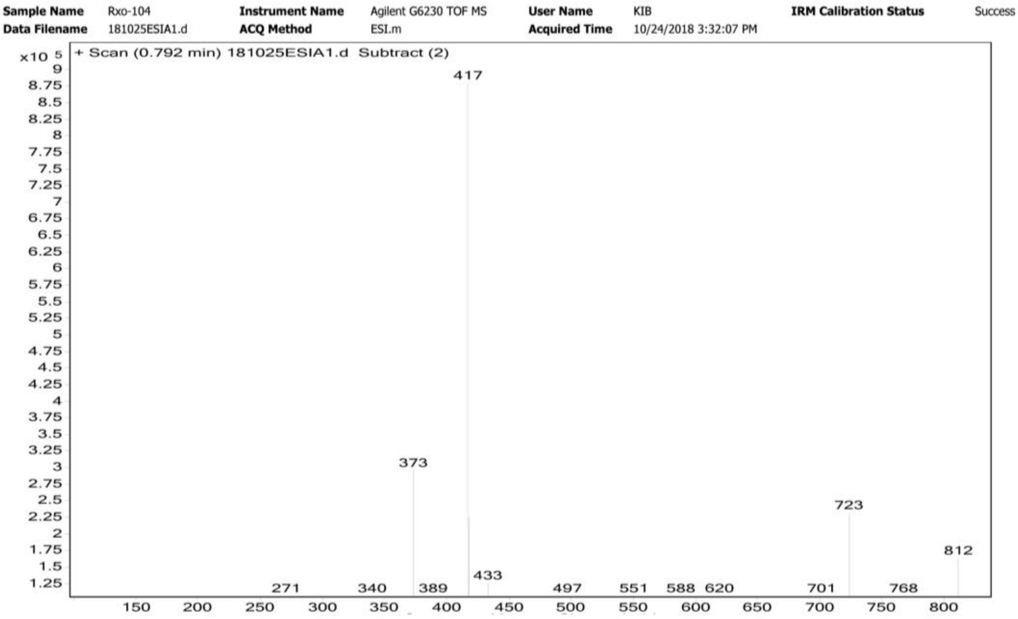

Figure S7. ESIMS spectrum of carascynol A (**1**).

Qualitative Analysis Report

**Data Filename**	181025ESIA1.d	**Sample Name**	Rxo-104
**Sample Type**	Sample	**Position**	
**Instrument Name**	Agilent G6230 TOF MS	**User Name**	KIB
**Acq Method**	ESI.m	**Acquired Time**	10/24/2018 3:32:07 PM
**IRM Calibration Status**	Success	**DA Method**	ESI.m
**Comment**			

Sample Group **Info.**
Acquisition SW 6200 series TOF/6500 series
Version Q-TOF B.05.01 (B5125.2)

User Spectra

Fragmentor Voltage	Collision Energy	Ionization Mode
200	0	ESI

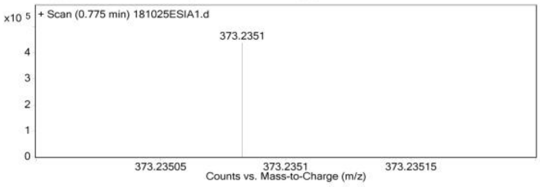

Peak List

m/z	z	Abund	Formula	Ion
340.2823	1	145318.66		
373.2351	1	438015.56	C21 H34 Na O4	M+
417.2972	1	1319470.63		
418.3009	1	340987.5		
433.2655	1	203095.8		
588.4096	1	203850.25		
701.4936	1	220503.75		
723.4805	1	313960.66		
811.6049	1	387495.22		
812.6079	1	195492.13		

Formula Calculator Element Limits

Element	Min	Max
C	0	200
H	0	400
O	0	10
Na	1	1

Formula Calculator Results

Formula	CalculatedMass	Mz	Diff.(mDa)	Diff. (ppm)	DBE
C21 H34 Na O4	373.2355	373.2351	0.4	1.0	4.5

--- End Of Report ---

Figure S8. HRESIMS spectrum of carascynol A (**1**).
